# Depression symptoms increase the risk for initiation or switching to biologic therapy in pediatric inflammatory bowel disease patients in remission

**DOI:** 10.1186/s12876-023-02993-z

**Published:** 2023-10-13

**Authors:** F. Milo, G. Angelino, E.F. Romeo, P. De Angelis, P. Tabarini

**Affiliations:** 1 Psychology Unit, Bambino Gesù Children’s Hospital, IRCCS, Rome, Italy; 2 Gastroenterology and Nutrition Unit, Bambino Gesù Children’s Hospital, IRCCS, Rome, Italy

**Keywords:** Pediatric, Inflammatory bowel disease, Depression, Anxiety, Biologic therapy

## Abstract

**Background and aims:**

Anxiety and depression symptoms are common in IBD population, both adult and pediatric patients. Increased psychological distress might contribute to initiation and switching to biologic therapy in adult patients with IBD or other chronic inflammatory diseases. Aim of the present study are to evaluate anxiety and depression symptoms in IBD pediatric patients with disease remission and investigate their role in initiation or switching to biologic therapy.

**Methods:**

We performed a retrospective analysis on IBD pediatric patients, assessing for anxiety (GAD-7) and depression (PHQ-9) symptoms. Demographic and disease characteristics were obtained from medical records.

**Results:**

Eighty-six patients [31 (36%) females - mean age = 15.6 (SD = 2.8) years] were included. Patients scored above cut-off (> 10) on PHQ-9 and GAD-7 were 17 (19.7%) and 18 (20.9%), respectively. No differences were found between UC and CD patients. Baseline clinically relevant depression symptoms were significantly associated with the odds of initiating or switching to biologic therapy within 2 years [OR = 4.5 (1.4–14.3)], even after confounders adjustment [4.2 (1.2–14.9)]. Relationship was not significant with anxiety symptoms.

**Conclusion:**

Anxiety and depression symptoms is relatively common in pediatric IBD population, even with disease remission. Pediatric IBD patients with high depression symptoms are at increased risk of initiating or switching to biologic therapy. Mental health screening programs should be incorporated in routine clinical practice, especially for depression, regardless of disease activity and disease type. Early diagnosis and proper intervention for mental illness should be part of routine IBD management.

## Introduction

Inflammatory bowel disease (IBD), comprising Crohn’s disease (CD), ulcerative colitis (UC), and inflammatory bowel disease-undefined (IBD-U), is characterized by chronic intestinal inflammation with a relapsing-remitting course. Main symptoms include diarrhea, abdominal pain, rectal bleeding/bloody stools, weight loss and fatigue [1]. The incidence and prevalence of pediatric IBD is increasing globally [2]. Pediatric age raises several age-specific considerations related to growth, pubertal development and the acquirement towards adulthood of autonomy, body image, and self-confidence [3]. Anxiety and depression symptoms are common in IBD patients, and disease activity has been described as an important risk factor for mental health both in adult and pediatric IBD population [4–7]. Conversely, psychological disorders such as anxiety or depression might be a risk factor for disease relapse, as demonstrated in adult IBD population [8].

Treatment options for pediatric IBD include non-biological therapies (such as aminosalicylates, thiopurines, and steroids) and biological therapies (monoclonal antibody targeting inflammatory cytokines) [9]. In a recent research on adult IBD population, elevated depressive symptoms were associated with two-fold increased odds of using biologic therapy [10]. Several studies on other chronic inflammatory conditions (such as Rheumatoid Arthritis) suggest that increased psychological distress symptoms may contribute to initiation and switching to biologic therapy [11, 12]. To date, no studies have investigated the association between anxiety and depression symptoms and step-up biologic therapy in IBD pediatric population. The aims of the present study are to evaluate anxiety and depression symptoms in IBD pediatric patients with disease remission and investigate their role in initiation or switching to biologic therapy.

## Methods

### Study design

We performed a retrospective analysis on data from consecutive IBD pediatric patients from Bambino Gesù Children’s Hospital of Rome, a tertiary care referral hospital with a dedicated pediatric IBD care program, caring for approximately 400 patients. Ethics approval from Bambino Gesù Ethics Committee was obtained for a comprehensive study about mental health assessment and therapeutic intervention in all IBD patients (protocol n. 2588). For the present study, retrospective data were collected from medical records of outpatient visits from January 1, 2019, to August 31, 2022. Demographic and disease variables were collected at baseline and after 12 months. A licensed psychologist examined psychological assessment through brief self-report instruments. Inclusion criteria were: (1) confirmed IBD diagnosis, (2) disease remission at baseline evaluation, (3) age > 11 years, (4) a sufficient knowledge of the Italian language to complete written questionnaires or clinical interview, and (5) minimum follow-up period of 12 months after psychological assessment. Exclusion criteria included active disease, neurological or neurodevelopmental impairment and/or other health conditions that could explain biologic initiation or switching therapy.

### Measures

Demographic and disease variables included the following: age, gender, body mass index (BMI), IBD duration, IBD type (UC or CD), surgical history, medications (including psychotropic drug, corticosteroid, mesalamine, immunomodulators and biologic therapy). Clinical indexes for disease activity, namely Pediatric Crohn’s Disease Activity Index (PCDAI) [13] and Pediatric Ulcerative Colitis Activity Index (PUCAI) [14], identified remission state (PUCAI or PCDAI score < 10 points). Biologic initiation or switching therapy was defined as any occurrence of infliximab, adalimumab, vedolizumab, or ustekinumab initiation or switching from one to other. Follow-up period was minimum 12 months and maximum 24 months after baseline assessment.

#### Generalized anxiety disorder (GAD-7)

The GAD-7 is a seven-item instrument that is used to measure or assess the severity of anxiety symptoms [15]. Items were scored on a four-point scale (0 = not at all, 1 = several days, 2 = more than half the days, and 3 = nearly every day), with total scores ranging from zero to twenty-one. The recommended screening cutoff was > 10, corresponding to at least a moderate level of anxiety [16].

#### Patient health questionnaire (PHQ-9)

The PHQ-9 is a depression symptoms scale consisting of nine questions and it can be used as a tool for monitoring the depressive symptoms. Items were scored on a four-point scale (0 = not at all, 1 = several days, 2 = more than half the days, and 3 = nearly every day), with total scores ranging from zero to twenty-seven. A score > 10 had a sensitivity of 89.5% and a specificity of 77.5% for detecting clinically meaningful depression symptoms in adolescents [17].

### Statistical analyses

Continuous variables were presented as mean ± standard deviation (SD) and categorical variables were presented as counts and percentage (%). Chi-squared test or Fisher exact test (as appropriate) was used to evaluate anxiety/depression symptoms differences between CD and UC. A binary logistic regression was used to investigate the effects of anxiety and depression on biologic initiation or switching therapy within the follow-up period (Unadjusted). A multiple logistic regression model was then performed, incorporating variables that have been previously associated with anxiety or depression as potential confounders (sex, age and disease type) [18], to examine the odds of biologic initiation or switching therapy with higher baseline anxiety and depression score (Adjusted). P values less than 0.05 were considered to be statistically significant. Data were analyzed using SPSS - Version 25 (Armonk, NY).

## Results

Out of 140 IBD patients aged 12–21 years who underwent psychological assessment during study period, 86 patients fulfilled the inclusion criteria for the study. Among them, 31 were female (36%, mean age = 15.6 ± 2.8 years). Forty-six (53%) had a diagnosis of UC and 40 (47%) were affected by CD. Sample characteristics are shown in Table 1.

**Table 1:** Sample characteristics

Baseline characteristic	Total (n = 86)		UC (n = 46)		CD (n = 40)	
Gender						
Female	31	36%	18	39%	13	32%
Male	55	64%	28	61%	27	68%
Age (years)	15.6	± 2.8	15	± 3	16	± 3
BMI (kg/m^2^)	20.3	± 5.5	21.2	± 5.5	19.4	± 5.5
Disease duration (months)	50	± 46	55	± 53	44	± 39
Medication						
Mesalamine	40	48.2%	26	56%	14	35%
Immunomodulator	14	16.9%	11	24%	3	7%
Oral corticosteroid	13	17.7%	9	19%	4	10%
Biologic	27	31.4%	10	22%	17	42%
Previous surgical resection	2	1.4%	1	2%	1	2%
Biologics initiation or switching therapy within 2y	36	41.9%	18	39%	18	45%
PHQ-9 > 10	17	19.7%	11	24%	6	15%
GAD-7 > 10	18	20.9%	10	22%	8	20%
PHQ-9 score	6.3	± 4.8	6.4	± 4.5	6.1	± 5.2
GAD-7 score	5.8	± 3.8	6.1	± 3.9	5.5	± 3.7
Previous psychotropic medication ^a^	6	7%	3	6%	3	7%

*Note*. N = 86. Data are expressed as counts and percentages for dichotomous variables and mean (± SD) for continuous variables. ^a^ Reflects the number and percentage of participants reported previous psychotropic medication, including antipsychotics (n = 2), mood stabilizer (n = 1), and anxiolytic (n = 3)

Average score for depression symptoms (PHQ-9) was 6.3 (SD = 4.8) and score for anxiety symptoms was 5.8 (SD = 3.8) (GAD-7). Patients with score above 10 on PHQ-9 and GAD-7 were 17 (19.7%) and 18 (20.9%), respectively. Concerning symptoms severity, approximately 50% of patients reported mild to severe symptoms, with no differences between UC and CD (Table 2). Binary logistic regression showed that baseline clinically meaningful depression symptoms (score > 10) were significantly associated with the odds of initiation or switching to biologic therapy within 2 years [OR = 4.5 (1.4–14.3)]. Anxiety did not show any influence on the risk to initiation or switching to biologic therapy [OR = 2.7 (0.9–7.8)]. In multiple logistic regression analysis, baseline depression increased the risk of biologic initiation or switching therapy [4.2 (1.2–14.9)], whereas baseline anxiety did not [2.3 (0.7–7.3)], after controlling for confounders. None of the confounders reached the statistical significance both for anxiety and depression model. Results of logistic regression are shown in Table 3.

**Table 2:** Severity of depression and anxiety symptoms according to disease type

Measure	UC			CD		*X* ^*2*^	p-value
	*N*	*%*		*N*	*%*		
*Depression symptoms (PHQ-9)*							
No symptoms	20	43.5		22	55	2.3	0.50
Mild	15	32.6		13	32.5		
Moderate	6	13		2	5		
Severe	5	11		3	7.5		
*Anxiety symptoms (GAD-7)*							
No symptoms	19	41		20	50	0.87	0.83
Mild	17	37		12	30		
Moderate	8	17.4		7	17.5		
Severe	2	4.3		1	2.5		

*Note.* X^2^ = chi-squared test of association; UC = Ulcerative colitis; CD Crohn’s Disease

**Table 3:** Logistic regression on the effect of baseline depression/anxiety and biologic initiation or switching therapy within 2 years

		OR	*95%CI*
*Unadjusted*	**Depression**	**4.5**	**1.4–14.3**
*Adjusted*	**Depression**	**4.2**	**1.2–14.9**
	Disease type (CD)	1.8	0.7–4.7
	Sex (male)	0.4	0.1–1.1
	Age (years)	0.9	0.7 − 1.0
*Unadjusted*	Anxiety	2.7	0.9–7.8
*Adjusted*	Anxiety	2.3	0.7–7.3
	Disease type (CD)	1.6	0.6–4.1
	Sex (male)	0.4	0.1–1.0
	Age (years)	0.6	0.7 − 1.0

*Note.* Number of patients = 86, CI = confidence interval. Bold type means statistically significance (p < .05)

## Discussion

Anxiety and depression symptoms are relatively common in IBD patients, compared to healthy subjects. Adult population seems to be more at risk than pediatric population, with a prevalence of 35–38% for anxiety and 20–24% for depression, compared to 16 and 15% in children, respectively [4, 5, 19]. Our study, including adolescents and youths, showed intermediate results, with a prevalence of 20.9% for anxiety and 19.7% for depression. No difference was noticed between UC and CD. These results are noteworthy, as they refer to patients with disease remission. This underlines the importance of mental health assessment in all pediatric IBD patients as a routine work up, regardless of disease activity and disease phenotype. Indeed, this approach could lead to early diagnosis of mental health symptoms and proper intervention.

Another intriguing result of the present study is that pediatric IBD patients with depression symptoms were four times more likely to start or switch to biologic therapy within two years, compared with non-depressed patients. These data are consistent with findings in adult population. For instance, a recent study demonstrated that IBD patients with elevated depression symptoms had two-fold times the odds of using biologic therapy [10]. In another IBD cohort, biologic medications were more frequently prescribed to patients with anxiety or depression symptoms [20]. The same trend has been observed in other chronic inflammatory diseases. Indeed, patients with Rheumatoid Arthritis and early depression were less likely to achieve a good response to biologic therapy [11, 21, 22]. Other Authors suggested that depression and anxiety might contribute to initiation and switching to biologics, either by aggravating disease severity and/or by distorting the perception of patient-reported outcome measures [12]. Depression symptoms were demonstrated to influence the course and severity of various chronic inflammatory diseases [23, 24]. Depressive symptoms might worsen the subjective experience of illness, decrease adherence to medications or limiting healthy behaviors, and have been associated with elevated levels of inflammation and inflammatory markers [25–27]. Based on our result and previous studies, we speculate that depression may contribute to worsening disease activity, reducing response to medication and leading to a step-up biologic therapy.

Limitations should also be noted. We did not capture the effect of several clinical factors [such as steroid dependence, disease duration, disease phenotype (stricturing/fistulizing) and BMI] can influence the risk of biologic usage and needs to be evaluated in multivariate analysis. We did not capture the use of psychological intervention or psychotropic therapies for depression and anxiety during the follow-up period; however, these therapies may influence the course of anxiety and depression symptoms and should be evaluated in the regression model.

## Conclusions

Clinically significant increase in anxiety and depression symptoms is relatively common in pediatric IBD population, even with disease remission. Prevalence seems to be equivalent in the two major disease phenotypes, namely UC and CD. Pediatric IBD patients with depression symptoms are more at risk of initiating or switching to biologic therapy, compared with patients without elevated depression symptoms. Mental health screening programs should be incorporated in routine clinical practice, especially for depression, regardless of disease activity and disease phenotype. Early diagnosis and proper intervention for mental illness should be part of routine IBD management.

## Data Availability

The datasets of this study are availability from the corresponding author on reasonable request.

## Abbreviations

IBD: Inflammatory Bowel Disease
CD: Crohn’s Disease
UC: Ulcerative Colitis
SD: Standard Deviation
BMI: Body mass index
